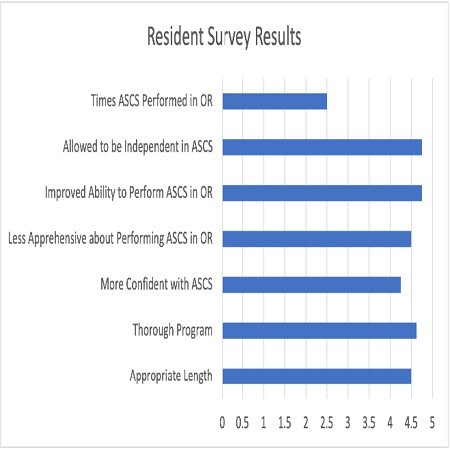# 760 Simulation Enhances Resident Performance Using Autologous Skin Cell Spray

**DOI:** 10.1093/jbcr/irae036.302

**Published:** 2024-04-17

**Authors:** Carl I Schulman, Shevonne S Satahoo, Louis R Pizano, Joyce I Kaufman

**Affiliations:** DeWitt Daughtry Family Department of Surgery, University of Miami, Miami, FL; DeWitt Daughtry Family Department of Surgery, University of Miami, Miami, FL; DeWitt Daughtry Family Department of Surgery, University of Miami, Miami, FL; DeWitt Daughtry Family Department of Surgery, University of Miami, Miami, FL

## Abstract

**Introduction:**

Surgical simulation has been shown to improve efficiency, performance, and shorten time to mastery for complicated operative procedures. Newer technologies used in the operating room can add to this complexity, especially in a training environment. Simulation training is not always considered when introducing a new medical device or product into the operating room. We hypothesized that simulation training would improve confidence and performance when using Autologous Skin Cell Spray (ASCS) in surgery residents during their burn rotation.

**Methods:**

The training was performed at the Surgical Training & Education Center at a large academic medical center. Residents were asked to read the instructional materials and watch the training videos before coming into the simulation lab. During the lab training, the resident performed each step of the process themselves while proctored by the burn attending and the simulation lab director. The process was allowed to proceed as slowly as necessary while answering all questions and providing feedback to the resident on their performance. A qualitative survey using a five-point Likert scale (strongly disagree, disagree, neutral, agree, strongly agree) was administered after their burn rotation.

**Results:**

Eight PGY3 residents completed the training and subsequently used ASCS in the OR. The survey evaluated their perceptions about the training program and its translation into performance in the OR. The program was felt to be of appropriate length (4.5/5) and thoroughness (4.625/5). There was very high agreement that the simulation training improved their confidence (4.25/5), independence (4.75/5) and performance (4.75/5) when using ASCS in the OR. Each resident used ASCS an average of 2.5 times during their 2-month rotation.

**Conclusions:**

Integrating simulation training improved self-reported confidence, independence, and performance for surgical residents using ASCS. Simulation training has the potential to decrease the time to proficiency and independent use of new technologies in the burn operating room. It may also increase efficiency and decrease operative time, especially in academic training programs. Further research is needed to quantify these real-world benefits.

**Applicability of Research to Practice:**

This study enhances our understanding of using simulation to enhance resident performance using complex new technologies on burn patients.